# A long-awaited book about fossil arachnids Book review: Dunlop JA, Penney D (2012) Fossil Arachnids. Monograph Series Volume 2. Siri Scientific Press, Manchester, 192 pp.

**DOI:** 10.3897/zookeys.183.3200

**Published:** 2012-04-19

**Authors:** Yuri M. Marusik, Mikhail M. Omelko

**Affiliations:** 1 Institute for Biological Problems of the North RAS, Portovaya Str. 18, Magadan, Russia; 2 Far Eastern Federal University, Sukhanova, 8, Vladivostok 690950 Russia; 3 Gornotaezhnaya Station FEB RAS, Gornotaezhnoe Vil., Ussuriyski Dist., Primorski Krai 692533 Russia

For a long time palaeoarthropodology has been ticking over in the background with publications mainly restricted to taxonomic papers in scientific journals. The deficit of more general overview works as a resource of background information for neontologists as well as palaeontologists was recently addressed for insects (Rasnitsyn and Quicke 2002; Grimaldi and Engel 2005). Now, for the first time this void has been filled for arachnids also, by two of the leading researchers in palaeoarachnology: Jason Dunlop (Germany) and David Penney (UK). Between them they have published more than 200 papers on the subject, including collaborative studies employing the latest cutting edge techniques (Dunlop et al. 2011a, b). The volume is dedicated to Professor Paul A. Selden (USA) who mentored both authors during the early stages of their academic careers. The volume opens with a very nice Rhynie palaeohabitat reconstruction by Richard Bizley (UK) showing that arachnids, in this case trigonotarbids, were amongst some of the first animals in early terrestrial ecosystems, approximately 410 million years ago. This is followed by a plate of drawings by J. Henry Blake showing the diversity of fossil spiders from the Tertiary Florissant deposits of North America, first published in 1890.

Following the dedication, foreword, a list of museum acronyms and a useful illustration of the geological timescale included for reference purposes, the work begins with an introduction to arachnids from a palaeontological perspective. This provides some historical references and then explains what arachnids are with regard to their anatomy, in order to provide the relevant information for what follows in the later chapters. This anatomical discussion is rather basic, but further order-specific details are provided later. The next of the introductory chapters concerns techniques for preparation and study of fossil arachnids. Here the authors allude to the different kinds of preservation seen in both amber and non-amber fossil deposits and how to extract the best morphological information out of the perserved arachnids, including the use of the latest techniques such as X-ray computed tomography. The authors do not go into detail here, but the work is fully referenced in order that the interested reader can pursue these subjects further if so desired.

The next chapter is unique in the arachnological literature to date and consists of a key to all 16 arachnid orders fossil and extant. This is a rather simple key focussing on features that are likely to be seen preserved in fossils and the basic body plan of each order is clearly illustrated to support the text. It is worth mentioning at this point that there are no other keys in the book and that it will not be of direct use in identifying fossils to family level and beyond, expect for a few rare instances, where a fossil specimen under investigation may correspond well to one of the photographs provided. This is unlikely to be the case for a spider in amber given their extreme diversity, but quite possible for a phalangiotarbid preserved in an ironstone nodule. The introductory section concludes with a discussion of the evolutionary relationships of the arachnids and closely related groups, which is nicely summarized in an evolutionary tree showing the hypothetical relationships and the known geological ranges of all orders. The tree is supported by a table of comparative diversity of fossil and extant species for each order. It should be noted that there are some discrepancies between these species richness numbers and those provided by Zhang (2011). However, the summary figures in Zhang (2011) do not add up correctly when the individual papers are examined. Given that the authors maintain a fossil arachnid database (Dunlop et al. 2012) that is updated every six months, it can be assumed that their numbers, at least for described fossil taxa, are the most accurate available.

Next follows the main content of the book, with chapters devoted to each arachnid order covered in detail. These include: Scorpiones, Opiliones, Phalangiotarbida, Palpigradi, Pseudoscorpiones, Solifugae, Acariformes, Parasitiformes, Ricinulei, Trigonotarbida, Uraraneida, Araneae, Haptopoda, Amblypygi, Thelyphonida and Schizomida. Each of these chapters follows a standardized format with an introduction followed by the following headed sections: Classification, Diagnostic characters, Descriptive characters (carapace, eyes, chelicerae, pedipalps, legs, opisthosoma, body size), Palaeodiversity (Palaeozoic, Mesozoic, Cenozoic), Fossil localities (Palaeozoic, Mesozoic, Cenozoic), Families recorded as fossils, Palaeoecology, and ending with a section on Important studies. The text is comprehensive, authoritative and fully referenced throughout, although more details and additional figures could have been devoted to the range of morphological variation of extant species within each order. The descriptive details provided do not do justice to the variation in seen in extant forms, although they should serve to facilitate identification of problematic arachnid compression fossils to order level. In addition, there could have been more in-depth coverage on the various systematic hypotheses that have been proposed (and are still unresolved) for some of the orders e.g. the mites and ticks, and even superfamilies within orders (e.g. Eresoidea in Araneae) although this may have tipped the arachnologist-palaeontologist-general biologist balance the book has aimed to achieve. Each chapter includes photographs of Recent species (for the extant orders) in order that non-arachnologists can contextualize the fossils. This section of the book is richly illustrated with large, photographs (mainly in colour) of both amber and non-amber fossils. The quality of the photographs is excellent and demonstrates the remarkable preservation of arachnids even in fossils dating back to the Carboniferous and beyond. Many of the fossils illustrated are types and repository data for all specimens illustrated are provided.

The final chapter, entitled Perspectives, summarizes what the authors hope to, and have achieved in the preceding pages. They also discuss how they expect palaeoarachnological research to develop in the future with particular regard to new fossils and new localities, the application of new imaging technologies and modern systematic methods, how palaeoarachnological data may be useful in modelling and predicting the consequences of tropical deforestation and global climate change, and the contribution that fossils can make to callibrating molecular clocks. The volume ends with an extensive bibliography and a taxonomic index to families and genera.

In terms of production, the book is of a high standard, well bound in a hard cover with end papers and printed on high quality, thick glossy paper meaning there is no show through from the reverse side of each page, although some may consider the margins a little too narrow. The text is of an easily readable appropriate size and has been very tightly copy edited. Scientific jargon has been kept to a minimum in order that the work can be more broadly accessible to non-academics. The photographs are large and very sharp, making the book a pleasure to the eye; even without the text the book would warrant a place in arachnological libraries purely based on the range and quality of the photographs, the majority of which have not been published elsewhere, at least not in colour and at such a large size.

In summary, we can highly recommend this book as absolutely unique within the arachnological literature to date. There is barely any overlap with previously published books on arachnids, which usually only briefly touch on the fossil record. It will fill a long-standing void on the shelves of arachnological libraries, and will be of interest to palaeontologists and neontologists alike, both as a source of reference or merely to browse through the stunning images it contains.
